# Primary Hyperoxaluria Type 1 in 18 Children: Genotyping and Outcome

**DOI:** 10.1155/2015/634175

**Published:** 2015-03-30

**Authors:** Mohamed S. Al Riyami, Badria Al Ghaithi, Nadia Al Hashmi, Naifain Al Kalbani

**Affiliations:** Pediatric Nephrology Unit, Department of Child Health, Royal Hospital, P.O. Box 1331, 111 Muscat, Oman

## Abstract

*Background*. Primary hyperoxaluria belongs to a group of rare metabolic disorders with autosomal recessive inheritance. It results from genetic mutations of the *AGXT* gene, which is more common due to higher consanguinity rates in the developing countries. Clinical features at presentation are heterogeneous even in children from the same family; this study was conducted to determine the clinical characteristics, type of *AGXT* mutation, and outcome in children diagnosed with PH1 at a tertiary referral center in Oman. *Method*. Retrospective review of children diagnosed with PH1 at a tertiary hospital in Oman from 2000 to 2013. *Result*. Total of 18 children were identified. Females composed 61% of the children with median presentation age of 7 months. Severe renal failure was initial presentation in 39% and 22% presented with nephrocalcinosis and/or renal calculi. Family screening diagnosed 39% of patients. Fifty percent of the children underwent hemodialysis. 28% of children underwent organ transplantation. The most common mutation found in Omani children was c.33-34insC mutation in the *AGXT* gene. *Conclusion*. Due to consanguinity, PH1 is a common cause of ESRD in Omani children. Genetic testing is recommended to help in family counseling and helps in decreasing the incidence and disease burden; it also could be utilized for premarital screening.

## 1. Introduction

Primary hyperoxaluria (PH) belongs to a group of rare metabolic disorders with autosomal recessive inheritance [1]. Type-1 PH (PH1) results from genetic mutations of the* AGXT* gene, which encodes the hepatic peroxisomal enzyme, alanine:glyoxylate-aminotransferase (AGT).* AGXT *is the only gene known to encode AGT and, thus far, approximately 146 mutations have been associated with PH1 [2–4]. In the absence of AGT activity, glyoxylate is converted to oxalate, and increased urinary oxalate excretion results in progressive intrarenal crystal deposition and kidney function deterioration [1]. The end result is systemic oxalosis [1]. The estimated prevalence of PH1 in Europe is 1–3 cases per million people [1, 5, 6], and the median age at initial presentation is 4 to 7 years old but ranges from the early neonatal period to the sixth decade of life [1, 6, 7]. Clinical features at presentation are heterogeneous and include recurrent hematuria, abdominal pain and urinary tract infections, nephrocalcinosis, nephrolithiasis, and even end-stage renal disease (ESRD), which hastens systemic oxalosis development [8]. However, PH1 can be a silent disease that is detected only during family screening [9]. The combination of a positive family history among siblings and cousins, ultrasonographic findings of nephrocalcinosis or nephrolithiasis, and suggestive laboratory features should alert nephrologists to the diagnosis. Conservative treatment measures should be initiated as soon as PH1 is suspected to prevent intrarenal crystal deposition and to slow progression to ESRD. These measures include high fluid intake, pyridoxine, and inhibitors of urinary crystallization such as citrate [1, 10].

This study was conducted to determine the clinical characteristics, type* AGXT* mutation, and outcome in children who were diagnosed to have PH1 at a tertiary referral center in Oman.

## 2. Methods

This study was a retrospective analysis of all Omani children <13 years old who were diagnosed with PH1 at the Royal Hospital, Muscat, Oman, from January 2000 to December 2013. Royal Hospital is the main tertiary referral center for children with kidney disease in Oman. Children included in this study were diagnosed with PH1 based on clinical features, family history, high urine oxalate, liver enzyme assay of AGT, or mutation analysis of the* AGXT* gene. The study was approved by the Royal Hospital Ethical Committee and written informed consent was obtained from a legal guardian.

Data collected from electronic medical records included sex, age, type of presentation, urinary oxalate findings, AGT activity results,* AGXT* gene analysis (if performed), type of conservative management received, age at ESRD development, type of dialysis received, and outcome (i.e., chronic kidney disease (CKD), transplant, or death).

Children with nephrocalcinosis or renal calculi that were suspected to have PH or were siblings of PH1 patients had blood investigations for renal function, renal ultrasound, and 24-hour urinary oxalate.

Hyperoxaluria was defined as urine oxalate >0.5 mmol/1.73 m^2^/day [11]. In children who presented with severe renal failure and had low urine oxalate level, plasma oxalate level was obtained.

To confirm the PH diagnosis and type, frozen liver samples from one patient in each family were evaluated for AGT activity at University College London Hospital, London, UK. Among the four families,* AGXT* mutation was analyzed in 7 patients at Center for Nephrology and Metabolic Disorder, Weisswasser, Germany.

Descriptive statistics were presented as percentages, means, and median.

## 3. Results

A total of 18 children from 4 different families were diagnosed with PH1 during the study period from January 2000 to December 2013.  Table 1 summarizes the main patient demographics. There were 61% females (11 out of 18 patients). Median age at presentation was 7 months (range 1–60 months). All children were below 5 years of age and 61% (11 out of 18 patients) were below 1 year of age at presentation. Nephrocalcinosis and/or renal calculi were common at presentation in 22% of our cohort (4 out of 18). Thirty-nine percent (7 out of 18) of patients presented with severe renal failure; majority were below the age of 1 year. In 4 out of these 18 children, the initial diagnosis of PH1 was not clear. Two patients were initially diagnosed to have renal tubular acidosis with nephrocalcinosis, but they later developed recurrent renal calculi and stone workup revealed elevated 24-hour urinary oxalate. The other two patients were initially diagnosed clinically as having autosomal recessive polycystic kidney disease. The first child presented with ESRD, started on peritoneal dialysis, and was only retrospectively diagnosed with PH1 after other siblings were diagnosed with PH1. The second child's diagnosis was reevaluated after developing recurrent renal calculi; stone workup showed high 24-hour urinary oxalate levels. 39% (7 out of 18) of the patients were diagnosed after family screening of confirmed PH1 siblings.

Supportive therapy such as citrate and pyridoxine was initiated in all patients (Table 2). High fluid intake was advised to patients who did not reach ESRD. Three children were at different stages of chronic kidney disease with current estimated GFR as shown in Table 2 under close follow-up. Renal replacement therapy in the form of intermittent hemodialysis (HD) was required for 50% of the children (9 out of 18). Peritoneal dialysis was performed in 3 infants who presented with renal failure and all of them died during follow-up. Two patients did not receive renal replacement therapy due to family refusal and later died due to systemic oxalosis. Three patients received liver transplantation only, while 2 patients received both liver and kidney transplantation. Patient IIa underwent renal transplantation twice; first renal graft was lost due to disease recurrence although liver transplant was working well. Fifty percent of patients with PH1 included in the study died.

Regarding liver biopsy result, AGT immunoreactivity was negative in two patients and AGT enzyme activity was low in all children who underwent liver biopsies (Table 3). Genetic testing after administrative approval was done in 7 patients among the 4 families. Five patients from 3 families had a c.33-34insC homozygous frame shift mutation in the* AGXT* gene. Two children from family IV had a C.346 G>A mutation in the* AGXT* gene as shown in Table 3. Consanguinity was confirmed in all four families.

## 4. Discussion

This is the first report of PH1 in Omani children. We identified 18 affected children from 4 different families, with each family having more than one affected child. Thus, the PH1 consanguinity rate in this study was 100%. There are many similar reports from different Middle East countries that showed elevated PH1 rates associated with increased consanguinity [12–15]. Mbarek et al. reported on 57 patients with PH1 from 40 different families. In their cohort, more than one family member was affected and the reported consanguinity rate was 75% [15]. In our study, all patients diagnosed with PH1 were <5 years old and the median age at initial PH1 symptom onset was 7 months. This age is lower compared to other reports. In the Netherlands, the median age at initial PH1 symptom onset is 6 years (range 0–50 years) [5], and in Japan the median age of onset was 13 years [16]. However, Mbarek et al. reported that the median age at presentation for patients carrying the 33-34insC mutation in Tunisian children was 3 years (range 5 months–61 years), while those children carrying the 1244T mutation were older, median age of 13 years (range 3 months–38 years) [15].

The clinical features at presentation in Omani PH1 patients were similar to those described in previous reports [5, 15, 17]. We found early-onset ESRD in our study; 39% (7 out of 18) presented with severe infantile PH1 and developed ESRD in the first year of life. Overall, the median age at ESRD development was about 4 years. In a study by van Woerden et al. [5] with a 7.7-year median follow-up, 33% of Dutch adult and child PH1 patients had ESRD at presentation and 16% developed ESRD during follow-up. While nine infants had infantile PH1, five of these patients developed ESRD, four out of these nine children were sensitive to pyridoxine, during follow-up three of them showed improved renal function or decreased nephrocalcinosis, and one had preserved renal function after pyridoxine administration.

A cohort of 222 PH1 patients from the Rare Kidney Stone Consortium (RKSC) registry showed renal survival rates of 89% at 10 years of age and 75% at 20 years of age [18]. We found two* AGXT* gene mutations in our patients: c.33-34insC in Families I, II, and III and a C.346 G>A mutation in Family IV. The 33-34insC mutation has been described before in many studies [15, 19, 20]; it is due to microinsertion on the major or minor alleles of the* AGXT* gene [21]. Children with this mutation in a homozygous state display no immunoreactive protein and no catalytic activity [22] compared to other mutations due to peroxisome-to-mitochondrial mistargeting. For example,* AGXT* mutations such as Gly170Arg and phe15Ile confer different degrees of catalytic activity and better outcomes because patients retain good responsiveness to pyridoxine [23, 24], whereas the previously reported 33-34insC mutation may retain only some responsiveness to pyridoxine [24, 25]. The C.346 G>A mutation is a missense mutation of* AGXT* gene; it was also described previously [20, 26]. However it is uncommon compared to 33-34insC mutation. In genetic analysis of 55 patients from USA using complete sequencing of the whole* AGXT* coding region, Monico et al. reported that the most common mutation in this cohort was G170R and then c.33-34inC but C.346 G>A was only found in one patient [20]. In our study, all children were very young at presentation and majority progressed rapidly to ESRD despite supportive measures including pyridoxine administration. However, confounding factors were therapy compliance issues and failure to regularly assess pyridoxine response.

van Woerden et al. [5, 19] previously conducted mutation analysis of 33 PH1 patients from a cohort of 57 patients and reported that the 33insC mutation was the third most common mutation and children who had this* AGXT* mutation in a homozygous state developed ESRD during early infancy with high mortality rate [19], similar to our findings. The results of the study by Mbarek et al. [15] are also in line with our findings. They identified the 33-34insC mutation as the second most common* AGXT* mutation, and children with this mutation display a severe form of PH1 with a high mortality rate. We noted no genotype-phenotype correlation between the two mutations in our cohort in view of similarities on severe early presentation and poor outcome. Our patients had severe disease with early presentation and genetic mutation was identified in at least one patient from each family. Therefore PHII is unlikely diagnosis among our cohort.

Regarding ESRD treatment, most of our patients received HD and a few were administered peritoneal dialysis. Many patients developed systemic oxalosis and this is attributed to multiple factors including the following. First, our patients had specific mutations that are associated with poor outcomes with multiple siblings affected within the family. However, previous reports [27, 28] showed poor correlation between genotype and phenotype, and even siblings with the same mutation can display different clinical features and have varied prognosis. Some studies have shown good outcomes with the homozygous 508G>A mutation compared to the 33insC mutation [19, 25]. Second, more intensive dialysis approaches such as daily HD or a combination of high flux HD and peritoneal dialysis were not routinely practiced in our institution. These more intensive dialysis approaches have better results compared to conventional dialysis, due to improved oxalate removal [7, 29]. The third and perhaps most important reason for the high mortality rate observed might be due to limited access to organ transplantation for both kidney and liver in our country; this observation is noted in other developing countries [15].

Five children in our study underwent organ transplantation. Two patients had living-related dual liver-kidney transplantation, two children with ESRD had living-unrelated liver transplants, and one patient with normal renal function had a preemptive living-related liver transplant. On follow-up, one patient died and the second child developed disease recurrence and required a second renal transplant. Hence, the overall survival rate in our cohort who underwent organs transplantation was 80%. European and American studies indicate that combined liver and kidney transplantation is the best currently available therapy for PH1 patients [29, 30]. The European registry reported that 1-, 5-, and 10-year patient survival rates were 86%, 80%, and 69%, respectively [30]. The US Renal Data System reported PH1 patient survival >80% at 5 years and a death-censored graft survival of 76% at 8 years after transplant [31]. We elected to do a preemptive liver transplant for one patient even though he displayed only nephrocalcinosis with normal renal function. This was decided with strong family support because he had confirmed genetic mutation and history of 2 siblings who died with the same disease. Preemptive isolated liver transplantation can be considered in selected cases but can raise ethical issues in view of PH1 heterogeneity in the progression of the disease and improvement on the conservative management of these patients in the last decade [7, 29].

## 5. Conclusion

PH1 is a common cause of ESRD in Omani children due to the high consanguinity rate. PH1 screening should be done in any child that presents with ESRD with unexplained reason. Genetic testing is recommended to determine the gene mutation which helps in family counseling and ultimately decreasing the incidence of this disease. Conservative measurements should be started immediately as they may delay the progression, with regular close monitoring for response. Children who progress to ESRD should have more aggressive dialysis and plan for early liver-kidney transplantation. This report is limited by being a retrospective study with a small sample size. Further studies are recommended to identify local gene mutations and help tailor therapies accordingly. In conclusion, this report is the first from Oman and the gulf region; it highlights the huge disease burden of an autosomal recessive mutation and importance of family screening and genetic counseling.

## Figures and Tables

**Table 1:** Demographic data and clinical features.

Number	Sex	Age at presentation (months)	Type of presentation	Age at onset of ESRD (years)

Ia	Male	18	NC, distal RTA, renal stone	11
Ib	Female	18	Family screening, NC	6
Ic	Male	2	ESRD	At presentation
IIa	Female	12	NC, RTA, renal stone	11
IIb	Female	7	ESRD	At presentation
IIc	Female	2	Family screening, NC	8
IId	Male	2	Family screening, NC, CKD	4
IIIa	Female	60	ESRD	At presentation
IIIb	Female	36	ESRD	At presentation
IIIC	Male	24	Family screening, NC	Normal RFT
IIId	Male	7	ESRD	At presentation
IVa	Female	6	NC, renal stone, UTI	7
IVb	Female	1	Family screening, NC	Below 1 year
IVc	Male	48	NC, polyuria	CKD
IVd	Female	1	Family screening, NC	CKD
IVe	Male	4	ESRD, NC	At presentation
IVf	Female	3	Family screening, NC	CKD
IVg	Female	2	ESRD, NC	At presentation

Family number 1 (I), family number 2 (II), family number 3 (III), and family number 4 (IV).

ESRD: end-stage renal disease, NC: nephrocalcinosis, RTA: renal tubular acidosis, RFT: renal function test, CKD: chronic kidney disease, and UTI: urinary tract infection.

**Table 2:** Management and outcome.

Number	Supportive therapy	Follow-up period (year)	RRT	Transplant	Outcome
Ia	C, F, P, ESWL	13	HD	—	Alive
Ib	C, F, P	7.5	HD	LUL	Alive
Ic	C, P	3.5	HD	—	Died
IIa	C, F, P, ESWL	13	HD	LRL and 2 LRK	Alive
IIb	C, P	2	PD	—	Died
IIc	C, F, P	9	HD	LRL and LRK	Alive
IId	C, F, P	3	—	—	Died
IIIa	C, P	4.5	HD	—	Died
IIIb	C, P	3.5	HD	—	Died
IIIc	C, F, P	4	—	LRL	Alive
IIId	C, P	2.5	HD	—	Alive
IVa	C, F, P, ESWL	9	HD	LUL	Died
IVb	C, P	1.5	—	—	Died
IVc	C, F, P	9	Supportive	—	^*^47
IVd	C, F, P	1.5	Supportive	—	^*^16
IVe	C, P	1	PD	—	Died
IVf	C, F, P	4	Supportive	—	^*^64
IVg	C, P	2	PD	—	Died

Family number 1 (I), family number 2 (II), family number 3 (III), and family number 4 (IV). ESRD: end-stage renal disease, C: citrate, F: fluid, P: pyridoxine, ESWL: electric shock wave lithotripsy, CKD: chronic kidney disease, HD: hemodialysis, PD: peritoneal dialysis, LRL: living-related liver transplant, LRK: living-related kidney transplant, LUL: living-unrelated liver transplant, and ^*^estimated glomerular filtration rate (eGFR mL/min/m²).

**Table 3:** 24-hour urinary oxalate, plasma oxalate, *AGXT* mutations and AGT activity.

Patient number	Urinary oxalate, mmol/24 h	Plasma oxalate, *μ*mol/L	*AGXT* mutation	AGT activity, *μ*mol/h/mg protein
Ia^*^	2.440	65	—	—
Ib	—	—	c.33-34insC	4.7
Ic^*^	0.228	—	—	—
IIa^*^	1.309	41	C.33-34insC	2.8
IIb	—	—	—	—
IIc^*^	0.331	27.7	C.33-34insC	—
IId^*^	0.224	58	—	—
IIIa	0.164	202	—	—
IIIb	0.052	109	—	2.4
IIIc^*^	1.567	—	C.33-34insC	—
IIId	0.076	27.0	C.33-34insC	—
IVa^*^	0.889	40	—	4.7
IVb^*^	0.526	—	c.346 G>A	—
IVc^*^	1.538	—	c.346 G>A	—
IVd^*^	0.165	—	—	—
IVe	0.021	127.0	—	—
IVf^*^	0.479	—	—	—
IVg	0.072	—	—	—

Family number 1 (I), family number 2 (II), family number 3 (III), and familynumber 4 (IV). ^*^24-hour oxalate >0.5 mmol/1.73 m^2^. Lab reference range for plasma oxalate <33 *μ*mol/L. AGT activity reference range (19.1–47.9 *μ*mol/h/mg); —: not done.

## References

[1] Harambat J., Fargue S., Bacchetta J., Acquaviva C., Cochat P. (2011). Primary hyperoxaluria. *International Journal of Nephrology*.

[2] Coulter-Mackie M. B., White C. T., Hurley R. M., Chew B. H., Lange D., Pagon R. A., Bird T. D., Dolan C. R., Stephens K., Adam M. P. (1993). Primary hyperoxaluria type 1. *Gene Reviews*.

[3] Robbiano A., Mandrile G., de Marchi M. (2010). Novel human pathological mutations. Gene symbol: AGXT. Disease: hyperoxaluria.. *Human genetics*.

[4] Williams E. L., Acquaviva C., Amoroso A. (2009). Primary hyperoxaluria type 1: update and additional mutation analysis of the AGXT gene. *Human Mutation*.

[5] van Woerden C. S., Groothoff J. W., Wanders R. J. A., Davin J.-C., Wijburg F. A. (2003). Primary hyperoxaluria type 1 in The Netherlands: prevalence and outcome. *Nephrology Dialysis Transplantation*.

[6] Cochat P., Deloraine A., Rotily M., Olive F., Liponski I., Deries N. (1995). Epidemiology of primary hyperoxaluria type 1. *Nephrology Dialysis Transplantation*.

[7] Cochat P., Liutkus A., Fargue S., Basmaison O., Ranchin B., Rolland M.-O. (2006). Primary hyperoxaluria type 1: still challenging!. *Pediatric Nephrology*.

[8] Hoppe B., Beck B. B., Milliner D. S. (2009). The primary hyperoxalurias. *Kidney International*.

[9] Hoppe B. (2010). Evidence of true genotype-phenotype correlation in primary hyperoxaluria type 1. *Kidney International*.

[10] Borghi L., Meschi T., Amato F., Briganti A., Novarini A., Giannini A. (1996). Urinary volume, water and recurrences in idiopathic calcium nephrolithiasis: a 5-year randomized prospective study. *The Journal of Urology*.

[11] Cochat P., Hulton S.-A., Acquaviva C. (2012). Primary hyperoxaluria Type 1: indications for screening and guidance for diagnosis and treatment. *Nephrology, Dialysis, Transplantation*.

[12] Madani K., Otoukesh H., Rastegar A., Van Why S. (2001). Chronic renal failure in Iranian children. *Pediatric Nephrology*.

[13] Kamoun A., Lakhoua R. (1996). End-stage renal disease of the Tunisian child: epidemiology, etiologies, and outcome. *Pediatric Nephrology*.

[14] Al-Eisa A., Naseef M., Al-Hamad N., Pinto R., Al-Shimeri N., Tahmaz M. (2005). Chronic renal failure in Kuwaiti children: an eight-year experience. *Pediatric Nephrology*.

[15] Mbarek I. B., Abroug S., Omezzine A. (2011). Selected *AGXT* gene mutations analysis provides a genetic diagnosis in28% of Tunisian patients with primary Hyperoxaluria. *BMC Nephrology*.

[16] Takayam T., Nagata M., Ichiyama I., Ozono S. (2005). Primary hyperoxaluria in Japan. *American Journal of Nephrology*.

[17] Hoppe B., Langman C. B. (2003). A United States survey on diagnosis, treatment, and outcome of primary hyperoxaluria. *Pediatric Nephrology*.

[18] Edvardsson V. O., Goldfarb D. S., Lieske J. C. (2013). Hereditary causes of kidney stones and chronic kidney disease. *Pediatric Nephrology*.

[19] Van Woerden C. S., Groothoff J. W., Wijburg F. A., Annink C., Wanders R. J. A., Waterham H. R. (2004). Clinical implication of mutation analysis in primary hyperoxaluria type1. *Kidney International*.

[20] Monico C. G., Rossetti S., Schwanz H. A. (2007). Comprehensive mutation screening in 55 probands with type 1 primary hyperoxaluria shows feasibility of a gene-based diagnosis. *Journal of the American Society of Nephrology*.

[21] Coulter-Mackie M. B., Applegarth D., Toone J. R., Henderson H. (2004). The major allele of the alanine:glyoxylate aminotransferase gene: seven novel mutations causing primary hyperoxaluria type 1. *Molecular Genetics and Metabolism*.

[22] Coulter-Mackie M. B., Rumsby G. (2004). Genetic heterogeneity in primary hyperoxaluria type 1: impact on diagnosis. *Molecular Genetics and Metabolism*.

[23] Cochat P., Rumsby G. (2013). Primary hyperoxaluria. *The New England Journal of Medicine*.

[24] Monico C. G., Rossetti S., Olson J. B., Milliner D. S. (2005). Pyridoxine effect in type I primary hyperoxaluria is associated with the most common mutant allele. *Kidney International*.

[25] Amoroso A., Pirulli D., Florian F. (2001). AGXT gene mutations and their influence on clinical heterogeneity of type 1 primary hyperoxaluria. *Journal of the American Society of Nephrology*.

[26] Amoroso A., Pirulli D., Puzzer D. (1999). Gene symbol: AGXT. Disease: primary hyperoxaluria type I. *Human Genetics*.

[27] Hoppe B., Danpure C. J., Rumsby G. (1997). A vertical (pseudodominant) pattern of inheritance in the autosomal recessive disease primary hyperoxaluria type 1: lack of relationship between genotype, enzymic phenotype, and disease severity. *American Journal of Kidney Diseases*.

[28] Leumann E., Hoppe B. (2005). Primary hyperoxaluria type 1: is genotyping clinically helpful?. *Pediatric Nephrology*.

[29] Cochat P., Groothoff J. (2013). Primary hyperoxaluria type 1: practical and ethical issues. *Pediatric Nephrology*.

[30] Jamieson N. V. (2005). A 20-year experience of combined liver/kidney transplantation for primary hyperoxaluria (PH1): the European PH1 transplant registry experience 1984–2004. *The American Journal of Nephrology*.

[31] Cibrik D. M., Kaplan B., Arndorfer J. A., Meier-Kriesche H.-U. (2002). Renal allograft survival in patients with oxalosis. *Transplantation*.

